# Effects of l-theanine–caffeine combination on sustained attention and inhibitory control among children with ADHD: a proof-of-concept neuroimaging RCT

**DOI:** 10.1038/s41598-020-70037-7

**Published:** 2020-08-04

**Authors:** Chanaka N. Kahathuduwa, Sarah Wakefield, Blake D. West, Jessica Blume, Tharaka L. Dassanayake, Vajira S. Weerasinghe, Ann Mastergeorge

**Affiliations:** 1 Department of Laboratory Sciences and Primary Care, School of Health Professions, Texas Tech University Health Sciences Center, 3601 4th Street, Lubbock, TX USA; 2 Department of Psychiatry, School of Medicine, Texas Tech University Health Sciences Center, 3601 4th Street, Lubbock, TX USA; 3 Department of Human Development and Family Studies, College of Human Sciences, Texas Tech University, Lubbock, TX USA; 4 Department of Physiology, Faculty of Medicine, University of Peradeniya, Peradeniya, Sri Lanka; 5 School of Psychology, The University of Newcastle, Callaghan, NSW Australia

**Keywords:** Paediatric research, Cognitive neuroscience, Attention, Cognitive control

## Abstract

We examined the acute effects of l-theanine, caffeine and their combination on sustained attention, inhibitory control and overall cognition in boys with attention deficit hyperactivity disorder (ADHD). l-Theanine (2.5 mg/kg), caffeine (2.0 mg/kg), their combination and a placebo were administered in a randomized four-way repeated-measures crossover with washout, to five boys (8–15 years) with ADHD. Functional magnetic resonance imaging (fMRI) was performed during a Go/NoGo task and a Stop-signal task ~ 1 h post-dose. NIH Cognition Toolbox was administered ~ 2 h post-dose. Treatment vs. placebo effects were examined in multi-level mixed-effects models. l-Theanine improved total cognition composite in NIH Cognition Toolbox (p = 0.040) vs. placebo. Caffeine worsened and l-theanine had a trend of worsening inhibitory control (i.e. increased Stop-signal reaction time; p = 0.031 and p = 0.053 respectively). l-Theanine–caffeine combination improved total cognition composite (p = 0.041), d-prime in the Go/NoGo task (p = 0.033) and showed a trend of improvement of inhibitory control (p = 0.080). l-Theanine–caffeine combination was associated with decreased task-related reactivity of a brain network associated with mind wandering (i.e. default mode network). l-Theanine–caffeine combination may be a potential therapeutic option for ADHD-associated impairments in sustained attention, inhibitory control and overall cognitive performance.

## Introduction

Attention deficit hyperactivity disorder (ADHD) is characterized by impaired attention, hyperactivity and increased impulsivity^1^. Impaired sustained attention, operationally defined as the inability to maintain voluntary focus on events that occur infrequently for a sustained period of time, is a major and extensively studied cognitive deficit associated with ADHD^2–4^. Similarly, multiple studies and meta-analyses have shown that impulse control is impaired in ADHD^5^. This repeatedly observed phenomenon led to the theory that lack of inhibitory control may be the primary cognitive deficit in ADHD^6^. In addition to impairments in sustained attention and impulse control, children with ADHD may also experience deficits in multiple executive functions including planning, visual and verbal working memory, and task-switching^2^. Thus, ADHD seems to affect multiple cognitive domains and these deficits may be contributing to academic underachievement that is associated with ADHD^7^.

Impaired sustained attention and inhibitory control in patients with ADHD is associated with increased task-associated mind wandering^8–12^. Increased mind wandering is negatively correlated with ADHD symptom severity^8,9^. Multiple functional MRI (fMRI) studies have established that a functional brain network, known as the default mode network (DMN), which includes the medial prefrontal cortex (anterior node of DMN), posterior cingulate cortex and precuneus (posterior node of DMN), shows increased reactivity during mind wandering even in neurotypical individuals^10^. Comparison of fMRI brain reactivity neurotypical vs. ADHD children while engaged in a sustained attention task revealed that children with ADHD show increased task-related reactivity of the posterior DMN^11^. In another study, high level of impulsivity was associated with decreased reactivity of both anterior and posterior DMN^12^. Thus, increased reactivity of DMN is an established phenomenon contributing to impaired sustained attention and inhibitory control among individuals with ADHD. In addition, functional neuroimaging surrogates of impaired inhibitory control in ADHD include decreased reactivity of several regions of a network known as the central executive network (CEN) (e.g. bilateral dorsolateral prefrontal cortex and inferior frontal gyrus)^13–15^ Accordingly, treatment of individuals with ADHD with stimulant medications (e.g. methylphenidate) acutely decreased the task-related hyperactivity of DMN while enhancing the reactivity of CEN^16,17^.

Even though several classes of medications are available to manage ADHD (e.g. stimulants such as methylphenidates and amphetamines; and non-stimulant medications such as atomoxetine, guanfacine and clonidine), intake of these medications are commonly associated with adverse effects ranging from loss of appetite, and irritability to suicidal ideation^18,19^ and therefore are not well-tolerated^20^. Moreover, poor responsiveness, pharmacological tolerance, risk of poisoning, high risk of misuse and improper use (e.g. despite contraindications) are common concerns of ADHD medications^21–23^. Therefore, there is an unmet need for safe, effective alternatives with known mechanisms of action to manage ADHD.

l-Theanine (l-γ-glutamylethylamide) and caffeine (1, 3, 7-trimethylxanthine) are two natural constituents of tea^24^. Caffeine has been known for decades to improve sustained attention in healthy adults (e.g. Refs.^25–27^, animal models with ADHD^28^ and even individuals with ADHD^29,30^. Furthermore, administration of caffeine was associated with an acute reduction of impulsivity in Spontaneous Hypertensive Rats, an animal model of ADHD^31^ and also the alcohol-associated impulsivity in healthy adults^32^. While there are hardly any animal studies on the effects of l-theanine on sustained attention or inhibitory control, our prior experience^26,33^ corroborates the findings of many others (e.g. Refs.^27,34^ to suggest that l-theanine improves sustained attention in healthy adults. Yet, some studies have indicated detrimental effects of l-theanine on reaction times and numerical working memory^27,35^ and of caffeine on inhibitory control^32^. However, the evidence regarding the effects of l-theanine–caffeine combination on sustained attention have been fairly consistent in suggesting the utility of both compounds in moderately improving measures of sustained attention in healthy adults^36^. Our prior studies conducted on healthy adults^26,33^, corroborate the findings of others^34,37^ to suggest that l-theanine–caffeine combination improves sustained attention in healthy adults, yet the relative contribution of l-theanine and caffeine to the improvements appear to largely depend on the task demands (e.g. difficulty, emphasis on accuracy vs. speed). We observed that administration of each of ~ 2.5 mg/kg body of l-theanine and ~ 2.0 mg/kg of caffeine as well as their combination improves performance in a visual sustained attention task^26^. In a subsequent functional magnetic resonance imaging (fMRI) study, this observation was replicated and both l-theanine and caffeine were found to decrease task-related reactivity of DMN, possibly decreasing mind wandering^33^. Therefore, l-theanine and caffeine seem to have a potential to be used to alleviate deficits of sustained attention in children with ADHD.

Safety and tolerability of l-theanine and caffeine have been extensively studied. Based on studies conducted in animal models, upon oral administration, both l-theanine and caffeine are rapidly absorbed from the small intestine^38^, increase in plasma in a dose-dependent manner and reach maximum plasma concentrations within 30 min of administration^39,40^. Plasma half-life of l-theanine ranges from 54–78 min and that of caffeine ranges from 2.5–4.5 h in human subjects^41,42^. Safety of oral administration of 400 mg/day of l-theanine (in two 200 mg doses) for 6 weeks to 49 boys (age 8–12 years) diagnosed with ADHD was established in a randomized clinical trial^43^. In this clinical trial, after 4 days of treatment with l-theanine, one child was documented to have developed facial tics, which subsided with discontinuation of treatment. No other adverse effects were noted. Similarly, administration of 3.0 mg/kg/day to children was not associated with any adverse events^44^. Moreover, when taken as a combination, l-theanine negated adverse effects of caffeine (e.g. sleep disturbances)^45^. Therefore, oral administration of a single dose of 2.5 mg/kg of l-theanine or 2.0 mg/kg of caffeine is very unlikely to result in an adverse event even among children.

Taken together, l-theanine, caffeine and their combination have been observed to improve sustained attention in healthy adults. Yet, the risk of development of adverse effects with low-moderate doses l-theanine or caffeine is minimal. Thus, l-theanine, caffeine and particularly their combination have the potential to be translated to manage cognitive deficits associated with ADHD. We aimed to examine the effects of l-theanine, caffeine and their combination on sustained attention, impulse control and overall cognition in male children with ADHD in a randomized placebo-controlled four-way repeated measures crossover study. We further aimed to concurrently explore the neurophysiological mechanisms of action of l-theanine, caffeine and their combination using fMRI, which provides a means of deducing neurophysiological processes that are associated with changes in cognition and behavior^46^. We hypothesized that l-theanine, caffeine and their combination would improve sustained attention and impulse control by decreasing task-related activity of DMN in the brain, which is thought to be associated with mind wandering. We further hypothesized that l-theanine, caffeine and their combination would improve impulse control by increasing reactivity of inferior frontal and dorsolateral prefrontal cortical regions of the brain that are thought to be the neural substrates of impulse control.

## Methods

### Participants

All procedures were conducted in accordace with the Helsinki declaration amended in 2000^47^. The protocol was approved by the Texas Tech University Human Research Protection Program. Informed written consent was obtained from all participants. The study protocol was pre-registered in ClinicalTrials.gov under the registry number NCT03533556 (https://clinicaltrials.gov/ct2/show/study/NCT03533556; 23/05/2018). Six male children (age 8–17 years) who have been diagnosed with ADHD by a psychiatrist or a pediatrician and have responded to stimulant medications were recruited via advertisement and referral. The sample was limited to males to avoid the influence of menstrual cyclical changes of hormones on reaction times^48^ and due to known differences in biochemical responses of the brains of males and females to caffeine, based on animal models of ADHD^49^. Including only males, while not ideal, allowed us to eliminate the effect of an important covariate (i.e. sex) in at this preliminary stage of the investigation. Based on the effect-size observed for the improvement of P300 cognitive event-related potential (i.e. a brain electrophysiological marker of attention) in a previous study conducted on healthy adults (d = 1.353)^26^, with six participants, the study was at least 75% powered to detect a significant improvement of sustained attention with l-theanine–caffeine combination as compared to placebo at a significance level of 0.05. While the sample size for this particular study was substantially small, given that four measurements were obtained from each participant for each outcome and as each participant acted as his own control in the repeated measures design, the study provided valuable data as ‘proof-of-concept’.

Potential participants/families expressing interest were subjected to a pre-screening telephone interview. Children with gross visual, hearing, intellectual, neurological or psychiatric impairments (except ADHD) that could affect performance in cognitive/neuropsychological test batteries were excluded. Similarly, children who were on medications except for stimulants that may affect cognitive functions and children who were on medications that may interact with caffeine were excluded. Given that the study involved a neuroimaging component, children with absolute contraindications to undergo magnetic resonance imaging were also excluded.

### Study design

The study was conducted as a randomized placebo-controlled four-way repeated measures crossover trial. All study visits were held at Texas Tech Neuroimaging Institute, Lubbock, TX. Each subject presented for an in-person screening visit and four testing visits scheduled at least 24 h apart to ensure adequate washout of the administered doses. During the in-person screening visit, the eligibility was confirmed via a structured interview and informed written consent (from a parent) and assent (from the child participants) were obtained. Then, Wechsler’s Intelligence Scale for Children 5th Edition (WISC-V); Pearson Education Inc., San Antonio, TX, USA) was administered using two iPads on the Q-interactive platform to screen for intellectual impairment (composite IQ < 80). WISC-V consists of a 1-h test battery, which has been normed for children 6–17 years to measure the fluid, verbal and composite IQ scores. Next, body weight was measured using an Omron HBF 514-C body composition monitor and scale (Omron Healthcare Inc., Lake Forest, IL, USA). Subsequently, 2.0 mg/kg body weight of caffeine was administered as a 100 ml solution to ensure that the participants could tolerate the bitter taste of caffeine. The participants were informed that the purpose of this administration is to ensure their ability to tolerate the taste of compounds that will be administered on certain testing visits. Finally, the participants were trained to perform a Go/NoGo continuous performance task and a Stop-signal task on a laptop computer and the four testing visits were scheduled.

**Figure 1:**
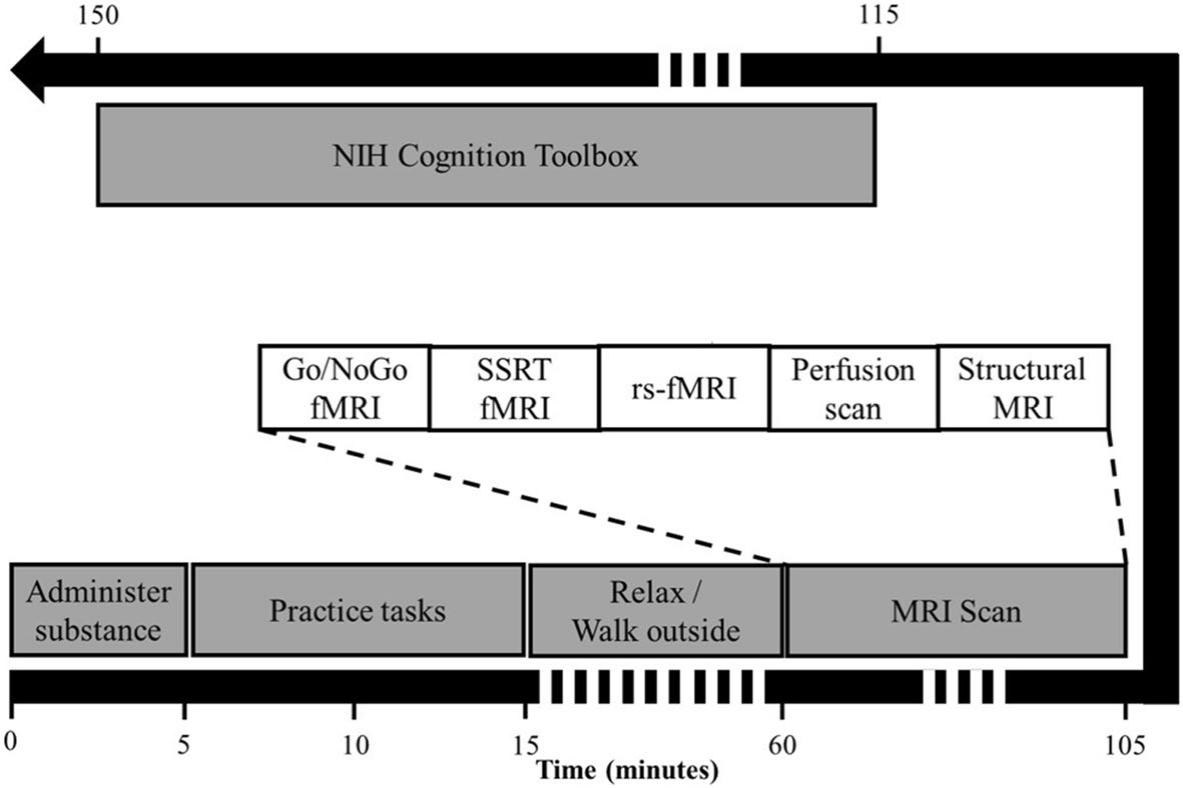
Data collection protocol.

The participants were instructed to sleep for at least 6 h and to avoid consumption of food or beverages that contain l-theanine or caffeine for at least 24 h before they presented for each testing visit. They were also instructed to refrain from taking stimulants medications for 24 h. Given the short half-life (3.5 h) and duration of therapeutic effect of the stimulant prescribed for the participants (i.e. methylphenidate hydrochloride)^50^, 24 h was deemed sufficient to avoid the effects of stimulants on the measured outcomes and to prevent potential interactions of stimulants with l-theanine or caffeine. On admission to each testing visit, a 100 ml solution that contained either (1) 2.5 mg/kg body weight of l-theanine (range 70–263 mg, (2 2.0 mg/kg body weight of caffeine (range 56–210 mg); (3) a combination of 2.5 mg/kg body weight of l-theanine and 2.0 mg/kg body weight of caffeine; or (4) none (i.e. 100 ml of water) were administered in a randomized counter balanced order (Fig. 1). The participants were expected to consume the 100 ml solution within 1 min. l-Theanine and caffeine were purchased in purified powder form (PureBulk Inc., Roseburg, OR, USA) to prepare the solutions. Appropriate doses for each participant was calculated based on the body weight measured during the screening visit and the doses were measured using a Gemini-20 millig scale with a precision of 1 mg (American Weight Scales Inc., Cumming, GA, USA). The solutions were made within 4 h of the time of administration by dissolving the doses of l-theanine and/or caffeine in a 100 ml aliquot of bottled water. The order of administration was determined using a pre-seeded random number generator in R statistical software (version 3.4.0; please see Supplementary Information 1) by CK. The participants were kept blind regarding the administered substances. Dose were administered by BW and JB. Given the bitter taste of caffeine and tasteless nature of l-theanine, each participant received two bitter-tasting and two tasteless solutions during the experiment.

The participants were allowed to practice the Go/NoGo task and the Stop-signal task for 10-min following administration of the substances. Then, the participants relaxed until 55 min had elapsed from the time of administration of the substance. Next, a scanning session was performed on a 3 T Siemens Skyra scanner equipped with a 20-channel head coil (see Supplementary Information 1 for the detailed scanning protocol including acquisition parameters). During the scanning session, first a 12.5-min functional scan was administered while the participants performed the Go/NoGo task. Second, another 12.5-min functional scan administered while the participants performed the Stop-signal task. The Go/NoGo task^51,52^ and the Stop-signal task^53^ were programed and presented using PsychoPy 2.0 (University of Nottingham, UK, see Supplementary Information 1 for the task description). The stimuli for the tasks were presented on an LCD screen and projected onto a mirror attached to the head coil of the scanner. The participants used a two-button fiber-optic hand-held device to respond to stimuli. The scanning session also included a 4.5-min T1-weighted structural scan.

After 110 min elapsed from the time of administration of the substances, the NIH Cognition Toolbox Test Battery (~ 30 min), which has been developed and normed for children and adults (3–85 years)^54,55^, was administered on an iPad. The battery provided an age-adjusted composite scores of overall cognition^56^ for each test session based on seven cognitive function tests: a flanker inhibitory control and attention test^57^, a picture sequence memory test^58^, a list sorting working memory test^59^, a picture vocabulary test^60^, an oral reading recognition test^60^, dimensional change card sort test^57^ and a pattern comparison processing speed test^61^.

### Data pre-processing and analysis

All behavioral data were analyzed via two-level models (i.e. testing visits nested within subjects) constructed using the lmerTest package in R statistical software (version 3.4.2). Each behavioral outcome was regressed on a dummy variable coded for the administered substance. Placebo condition was included in all models as the reference category. Testing visit number was included in all models as a covariate to account for the practice effect. In the Go/NoGo task, rate of correctly-responded Go trials (i.e. hit rate), rate of responded NoGo trials (i.e. false alarm rate), sensitivity to the Go signal (i.e. d-prime) computed based on the signal detection theory^62,63^, and reaction time to Go trials were regressed on the administered substance. In the Stop-signal task, mean reaction time to correctly-responded Go trials within a testing visit, rate of correctly-inhibited Stop trials (i.e. inhibition rate), mean Stop-signal delay and Stop-signal reaction time (SSRT) computed based on the horse-race model^64^ were considered as outcome variables. Age-adjusted total cognition composite scores obtained from NIH Cognition Toolbox Test Battery were similarly regressed on the administered substance.

Raw structural and functional MRI data were converted to NIfTI format using dcm2nii converter^65^ and were pre-processed using tools in Freesurfer^66,67^ and FSL (version 6.0, Oxford, UK) software (see Supplementary Information 1 for details on pre-processing). Functional MRI data of the Go/NoGo and Stop-signal tasks were analyzed via two-level models constructed using the FEAT tool in FSL (see Supplementary Information 1 for the detailed analysis protocol). In level 1 analyses, contrasts were modeled to examine the brain reactivity to correctly-responded Go trials and correctly-inhibited NoGo trials in the Go/NoGo task and inhibitory control in the Stop-signal task. In level 2 analyses, level 1 contrasts for each treatment was compared against the placebo within DMN and CEN, maintaining FWER at 0.05 via a permutation-based cluster thresholding approach.

## Results

### Demographic information

**Figure 2:**
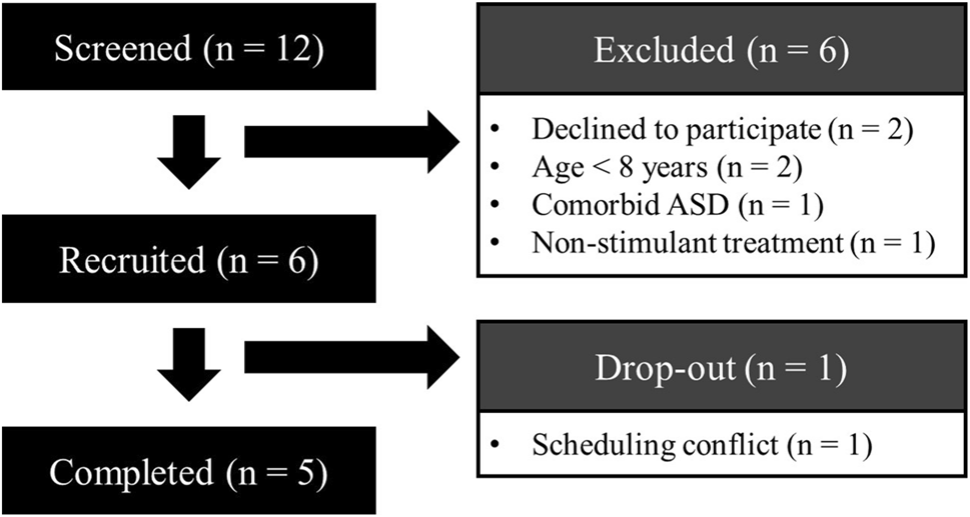
CONSORT diagram outlining the details of participant screening, recruitment and completion of data collection.

Five enrolled participants completed the study (see Fig. 2 for the CONSORT diagram) during the data collection period of 14/05/2018–31/08/2018 and was ended due to expiration of funding. One consented participant declined to participate prior to administration of the first test dose due to a scheduling conflict. Thus, only the baseline data and outcomes of the five completers were analyzed. Mean age of the five participants who completed the study protocol was 11.87 ± 2.26 years (range 9.50–15.17). Body weights of the participants ranged from 27.95 to 105.23 kg (mean 60.86 ± 31.57) and the body mass index ranged from 14–94–33.29 kg m^−2^ (mean 23.75 ± 7.78). Mean verbal comprehension index and fluid reasoning index of the participants were 100.20 ± 9.33 (range 92–116) and 101.80 ± 14.17 (range 88–121) respectively, while the mean full-scale IQ as measured by the Wechsler’s Intelligence Scale for Children (5th Edition) was 99.80 ± 15.48 (range 85–122).

### Prescribed medications

Four participants were not on stimulant medications during the period in which the testing visits were scheduled (i.e. summer vacation), yet all participants reported symptomatic benefits of taking methylphenidate hydrochloride during the school year. One participant was on a methylphenidate hydrochloride daily (PRN) dose during the period of testing and reported having treatment free days during school vacation as directed by his physician. As such, the participant or his parents did not complain of adverse effects of withholding methylphenidate prior to testing sessions. Even though the participants were allowed to take stimulant medications upon completion of testing sessions, participants or parents did not complain of any symptoms suggestive of adverse effects of l-theanine/caffeine treatment or potential interactions between l-theanine or caffeine with methylphenidate.

### NIH cognition toolbox

Regression coefficients of the two-level regression models constructed to predict the outcomes of the NIH Cognition Toolbox Test Battery, Go/NoGo task and Stop-signal task compared the effects of each administered substance against the placebo on the tested outcome variables when controlled for the practice effect (Table 1). Mean age-adjusted cognition composite of the NIH Cognition Toolbox Test Battery following administration of l-theanine and l-theanine–caffeine combination were respectively 11.5 and 11.4 points greater than the mean cognition composite score achieved following administration of placebo (p = 0.040, Cohen’s d = 1.041 and p = 0.041, Cohen’s d = 1.034 respectively). While administration of caffeine was also associated with a mean improvement of the total cognition composite by 7.70 compared to placebo, this mean difference was not statistically significant (p = 0.148, Cohen’s d = 0.697).

**Table 1:** Results of multi-level regression models that compared the effects of administration of l-theanine, caffeine and their combination against a placebo, controlling for the testing visit to account for practice effect (N = 5, four repeated measurements per participant; 20 total measurements per outcome).

Dependent variable	Predictor	Estimate	SE	df	t	p
Cognition composite	Intercept	80.86	8.38	8.88	9.647	< 0.001***
	l-Theanine	11.50	4.94	11.00	2.326	0.040*
	Caffeine	7.70	4.94	11.00	1.558	0.148
	T + C	11.40	4.93	11.00	2.310	0.041*
	Visit	5.52	1.57	11.00	3.521	0.005**
Go/NoGo task Go reaction time	Intercept	0.38	0.01	14.24	31.708	< 0.001***
	l-Theanine	0.00	0.01	11.00	0.376	0.714
	Caffeine	− 0.02	0.01	11.00	− 1.750	0.108
	T + C	− 0.01	0.01	11.00	− 0.771	0.457
	Visit	− 0.01	0.00	11.00	− 2.120	0.058
Go/NoGo task Hit rate	Intercept	0.58	0.09	6.48	6.487	< 0.001***
	l-Theanine	0.21	0.04	11.00	5.045	< 0.001***
	Caffeine	0.23	0.04	11.00	5.447	< 0.001***
	T + C	0.24	0.04	11.00	5.772	< 0.001***
	Visit	− 0.04	0.01	11.00	− 2.830	0.016*
Go/NoGo task False alarm rate	Intercept	0.08	0.02	14.60	3.360	0.004**
	l-Theanine	0.01	0.02	11.00	0.540	0.600
	Caffeine	− 0.01	0.02	11.00	− 0.237	0.817
	T + C	− 0.01	0.02	11.00	− 0.434	0.673
	Visit	− 0.01	0.01	11.00	− 0.989	0.344
Go/NoGo task d-prime (sensitivity to signal)	Intercept	1.68	0.34	14.33	4.918	< 0.001***
	l-Theanine	0.38	0.27	11.00	1.411	0.186
	Caffeine	0.56	0.27	11.00	2.124	0.057
	T + C	0.65	0.27	11.00	2.436	0.033*
	Visit	− 0.05	0.08	11.00	− 0.598	0.562
Stop-signal task Go reaction time	Intercept	0.52	0.02	13.92	21.658	< 0.001***
	l-Theanine	0.00	0.02	11.00	0.130	0.899
	Caffeine	0.00	0.02	11.00	− 0.204	0.842
	T + C	− 0.04	0.02	11.00	− 1.662	0.125
	Visit	0.01	0.01	11.00	1.211	0.251
Stop-signal task Inhibition rate	Intercept	0.58	0.06	13.60	9.640	< 0.001***
	l-Theanine	− 0.09	0.05	11.00	− 1.974	0.074
	Caffeine	− 0.12	0.05	11.00	− 2.566	0.026*
	T + C	− 0.06	0.04	11.00	− 1.260	0.234
	Visit	0.02	0.01	11.00	1.544	0.151
Stop-signal delay	Intercept	0.35	0.03	14.12	13.592	< 0.001***
	l-Theanine	− 0.04	0.02	11.00	− 1.939	0.079
	Caffeine	− 0.05	0.02	11.00	− 2.605	0.024*
	T + C	0.00	0.02	11.00	0.050	0.961
	Visit	0.01	0.01	11.00	1.963	0.075
Stop-signal reaction time	Intercept	0.17	0.03	11.67	6.002	< 0.001***
	l-Theanine	0.04	0.02	11.00	2.165	0.053
	Caffeine	0.05	0.02	11.00	2.481	0.031*
	T + C	− 0.04	0.02	11.00	− 1.930	0.080
	Visit	0.00	0.01	11.00	− 0.674	0.514

### Go/NoGo task

In the Go/NoGo task, compared to the placebo, l-theanine–caffeine combination significantly improved the sensitivity to the Go signal (i.e. d-prime) in the Go/NoGo task computed based on the signal detection theory (β = 0.65, p = 0.033, Cohen’s d = 1.077), while caffeine was associated with a trend of improvement (β = 0.56, p = 0.057, Cohen’s d = 0.928) (Table 1). Intake of l-theanine did not significantly improve sensitivity to the Go signal (β = 0.38, p = 0.186, Cohen’s d = 0.629). On an exploratory factorial multi-level regression analysis, the main effects of l-theanine and caffeine factors remained the same, however, the interaction term was not significant (β = − 0.29, p = 0.450), suggesting that the effects of l-theanine and caffeine on improving d-prime in a Go/NoGo task may be additive (Table 2). l-Theanine, caffeine and their combination significantly improved Go/NoGo hit rate (Table 1), but did not have a significant effect on the reaction times or the false alarm rates in the Go/NoGo task.

**Table 2:** Results of factorial multi-level regression models that explored the interactive effects of l-theanine and caffeine when administered as a combination, controlling for the testing visit to account for practice effect (N = 5, four repeated measurements per participant; 20 total measurements per outcome).

Dependent variable	Predictor	Estimate	SE	df	t	p
Go/NoGo task Hit rate	Intercept	0.58	0.09	6.48	6.487	< 0.001***
	l-Theanine	0.21	0.04	11.00	5.045	< 0.001***
	Caffeine	0.23	0.04	11.00	5.447	< 0.001***
	Interaction	− 0.04	0.01	11.00	− 2.83	0.007**
	Visit	− 0.2	0.06	11.00	− 3.339	0.016*
Go/NoGo task d-prime (sensitivity to signal)	Intercept	1.68	0.34	14.33	4.918	< 0.001***
	l-Theanine	0.38	0.27	11.00	1.411	0.186
	Caffeine	0.56	0.27	11.00	2.124	0.057
	Interaction	− 0.05	0.08	11.00	− 0.598	0.452
	Visit	− 0.29	0.38	11.00	− 0.78	0.562
Stop-signal reaction time	Intercept	0.17	0.03	11.67	6.002	< 0.001***
	l-Theanine	0.04	0.02	11.00	2.165	0.053
	Caffeine	0.05	0.02	11.00	2.481	0.031*
	Interaction	0.00	0.01	11.00	− 0.674	0.001**
	Visit	− 0.13	0.03	11.00	− 4.637	0.514

### Stop-signal task

Similarly, in the Stop-signal task, oral administration of l-theanine, caffeine or their combination did not significantly change the reaction times to the Go stimuli compared to placebo. However, administration of caffeine was associated with decreased inhibitory control as evidenced by a significantly increased SSRT (β = 0.05, p = 0.031) compared to placebo. Furthermore, l-theanine was also associated with a trend of increased SSRT compared to placebo (β = 0.04, p = 0.053) that approached significance. Interestingly, l-theanine–caffeine combination was associated with a trend of improvement (i.e. a trend of increase) of inhibitory control as evidenced by a reduction of SSRT compared to placebo that approached significance (β = -0.04, p = 0.080, Cohen’s d = 0.894). On a factorial multi-level regression analysis that aimed to explore the interactive effects of l-theanine and caffeine when administered as a combination on the SSRT, the main effects of l-theanine and caffeine factors remained the same, however, a negative significant interaction was observed (β = -0.13, p < 0.001). This finding suggested that when administered in combination, l-theanine and caffeine counteract the adverse effect of reduction of inhibitory control and result in an overall improvement in inhibitory control.

### Brain fMRI reactivity to Go/NoGo task

**Figure 3:**
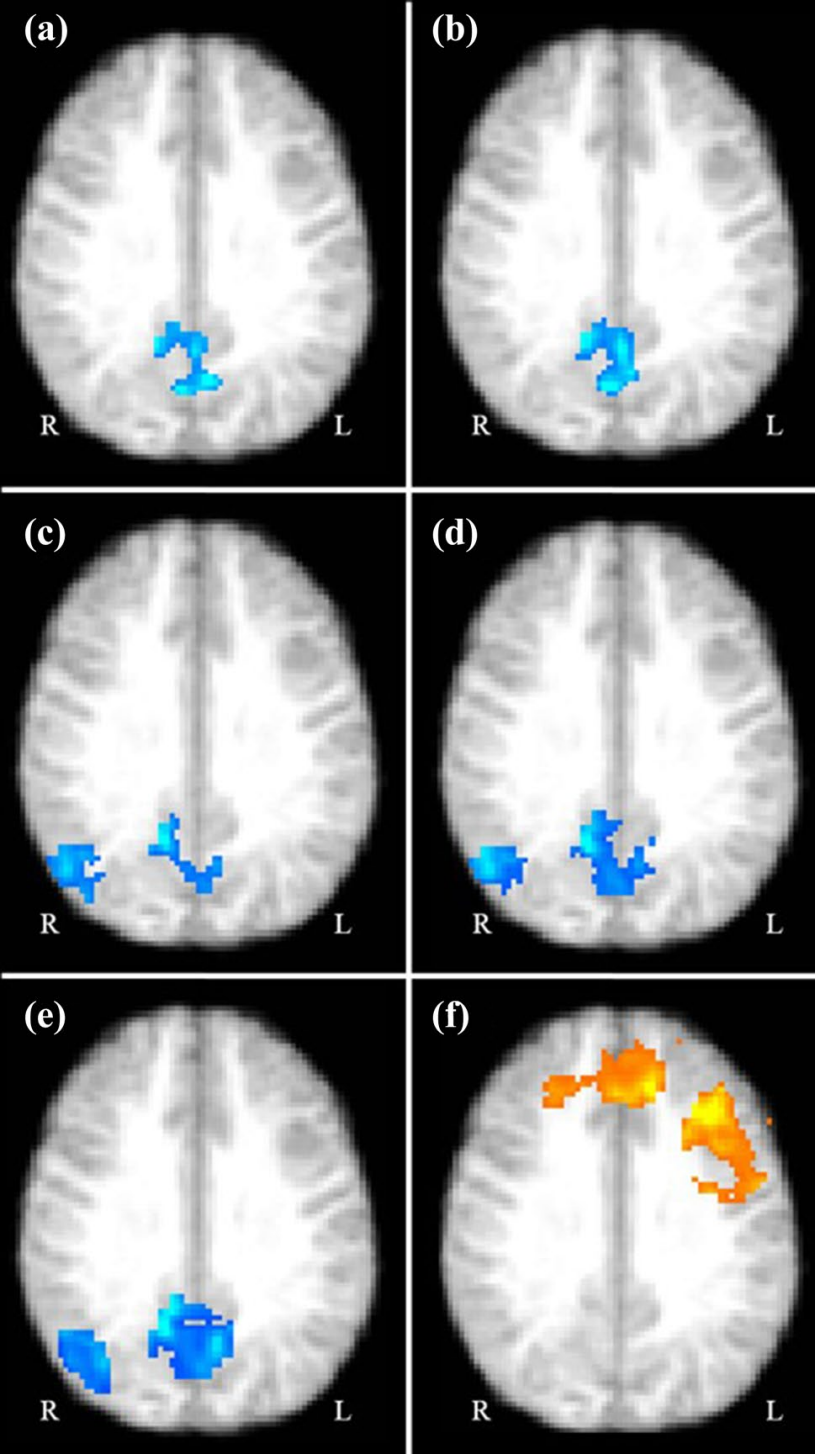
Brain regions that showed significant task-related fMRI reactivity following administration of l-theanine, caffeine and their combination vs. placebo, controlling for the testing visit to account for practice effect (N = 5, four repeated measurements per participant; 20 total measurements per outcome). (**a**) A cluster within DMN showing significant decreased reactivity when exposed to correctly responded Go stimuli in the Go/NoGo task following intake of l-theanine vs. placebo. (b) A cluster within DMN showing significant decreased reactivity when exposed to correctly responded Go stimuli in the Go/NoGo task following intake of caffeine vs. placebo. (**c**) A cluster within DMN showing significant decreased reactivity during response inhibition while engaged in the Stop-signal reaction task following intake of l-theanine vs. placebo. (**d**) A cluster within DMN showing significant decreased reactivity during response inhibition while engaged in the Stop-signal reaction task following intake of caffeine vs. placebo. (**e**) A cluster within DMN showing significant decreased reactivity during response inhibition while engaged in the Stop-signal reaction task following intake of the combination of l-theanine and caffeine vs. placebo. (**f**) A cluster within CEN showing significant decreased reactivity during response inhibition while engaged in the Stop-signal reaction task following intake of the combination of l-theanine and caffeine vs. placebo. (**g**) *DMN* default mode network, *CEN* central executive network.

On analysis of fMRI data of the Go/NoGo task, administration of l-theanine and caffeine were observed to significantly decrease reactivity of the posterior DMN (i.e. bilateral precuneus cortex, posterior cingulate cortex and cuneus cortex) vs. placebo (p = 0.034 and p = 0.026 respectively) (Fig. 3; Table 3). Given that greater reactivity of the posterior DMN is associated with mind-wandering, these could be considered as supportive evidence of decreased mind-wandering with l-theanine and caffeine when a child with ADHD engages in processing Go stimuli. However, administration of l-theanine–caffeine combination was not associated with a significant change in activity of DMN. l-theanine, caffeine or their combination was not associated with a significant change in the activity of CEN compared to placebo. Similarly, significant differences in brain reactivity within the DMN or CEN were not observed with correctly inhibited NoGo trials and false alarms in the Go/NoGo task. Taken together, l-theanine and caffeine seem to improve hit rate in the Go/NoGo task, possibly by decreasing mind wandering. Yet, neurophysiological mechanism underlying the greater improvements of the hit rate observed with l-theanine–caffeine combination was not elucidated.

**Table 3:** Brain regions that showed significant task-related fMRI reactivity following administration of l-theanine, caffeine and their combination compared to a placebo (N = 5, four repeated measurements per participant; 20 total measurements per outcome).

Task	Contrast	Substance	Network	Reactivity	Cluster size	Peak MNI coordinates			Brain Regions	p
						X	Y	Z		
Go/NoGo	Correct hits	l-Theanine vs. placebo	DMN	Decrease	909	8	− 40	12	bilateral precuneus	0.034
									bilateral posterior cingulate cortex	
Go/NoGo	Correct hits	Caffeine vs. placebo	DMN	Decrease	1,014	− 4	− 62	12	bilateral precuneus	0.026
									bilateral posterior cingulate cortex	
SSRT	Response inhibition	l-Theanine vs. placebo	DMN	Decrease	1,054	− 10	− 44	− 6	L/lingual gyrus	0.022
									L/precuneus	
									L/posterior cingulate cortex	
				Decrease	947	60	− 58	10	R/lateral occipital cortex	0.029
									R/precuneus	
									R/posterior cingulate cortex	
SSRT	Response inhibition	Caffeine vs. placebo	DMN	Decrease	3,084	32	− 36	− 22	R/temporal fusiform cortex	0.003
									R/parahippocampal gyrus	
									R/precuneus	
									R/posterior cingulate cortex	
					1,083	42	− 64	10	L/lateral occipital cortex	0.034
									L/precuneus	
									L/poosterior cingulate cortex	
SSRT	Response inhibition	l-Theanine–caffeine combination vs. placebo	DMN	Decrease	4,079	14	− 60	0	L/lingual gyrus	0.002
									L/lateral occipital cortex	
									bilateral precuneus	
									bilateral posterior cingulate cortex	
SSRT	Response inhibition	l-Theanine–caffeine combination vs. placebo	CEN	Decrease	7,272	− 36	26	− 20	L/frontal orbital cortex	0.007
									L/inferior frontal gyrus (triangularis)	
									bilateral middle frontal gyrus	
									bilateral superior frontal gyrus	
									bilateral precentral gyrus	

### Brain fMRI reactivity to Stop-signal task

While exerting inhibitory control in the Stop-signal task, the reactivity of the posterior DMN (i.e. bilateral precuneus, posterior cingulate cortex, right angular gyrus and right lateral occipital cortex) was significantly decreased by l-theanine, caffeine and their combination compared to placebo (p = 0.022, p = 0.003 and p = 0.002 respectively). This finding suggested that intake of l-theanine, caffeine and their combination may be associated with decreased mind wandering while engaged in an inhibitory control task. However, l-theanine or caffeine did not significantly affect reactivity of CEN compared to placebo. Interestingly, administration of the l-theanine–caffeine combination was associated with a significant decrease in reactivity of CEN compared to placebo in a cluster that included the bilateral middle and superior frontal, paracingulate and precentral gyri and the left inferior frontal gyrus (p = 0.007). These findings indicated that decreased fMRI reactivity of the bilateral anterior CEN, but not decreased reactivity of DMN, may be the neurophysiological mechanism through which the l-theanine–caffeine combination improves inhibitory control while engaged in the Stop-signal task.

## Discussion

Even though the beneficial effects of l-theanine, caffeine and their combination on sustained attention in healthy adults were known, the applicability of especially l-theanine and l-theanine–caffeine combination to improve sustained attention in children with ADHD was not established. In this novel study, we examined the effects of l-theanine, caffeine and their combination on not only sustained attention, but also inhibitory control and overall cognition as determined by the NIH Cognition Toolbox in children with ADHD. We further explored the neurophysiological mechanisms through which l-theanine, caffeine and their combination seem to affect sustained attention and inhibitory control in children with ADHD. Compared to the placebo, l-theanine significantly improved total cognition composite and sustained attention (i.e. Go/NoGo hit rate) and had a trend in decreasing inhibitory control as measured by Stop-signal reaction time. Similarly, caffeine improved Go/NoGo hit rate relative to the placebo and worsened inhibitory control as determined by increased Stop-signal reaction time and decreased inhibition rate. l-theanine–caffeine combination improved cognition composite, improved sustained attention as determined by hit rate as well as sensitivity to the Go signal (i.e. d-prime) in the Go/NoGo task and showed a trend of improvement of inhibitory control in the Stop-signal task. All substances decreased task-related brain reactivity of the DMN compared to the placebo, suggesting decreased task-related mind wandering.

Caffeine has been shown to improve attention deficit in animal models of ADHD^28,68^. Moreover, in a double-blind crossover study, Reichard and Elder observed significant improvements of accuracy in a choice reaction time task administered to hyperkinetic children with the intake of 6.0 mg/kg body weight of caffeine^29^. Similarly, following a double-blind crossover trial, Garfinkel et al. reported that intake of a low dose of caffeine by 6–10 years old children with attention deficit disorder experienced a significant improvement of teacher-reported behavior^30^. The significant improvement of the hit rate in the Go/NoGo task we observed with caffeine as compared to the placebo corroborates the findings of these previous studies to suggest that intake of caffeine may acutely improve sustained attention in children with ADHD. In addition, our study suggested that children with ADHD may experience improvements in sustained attention, as evidenced by improved hit rates in the Go/NoGo task, with the intake of l-theanine as well as the combination of l-theanine and caffeine. Factorial regression analyses conducted to examine the interactive effects of l-theanine and caffeine when administered in combination revealed a negative interaction of l-theanine and caffeine factors in the sustained attention task. This negative interaction suggests that administration of the two substances as a combination gives rise to a less potent action than what is expected to see with addition of the effects of the two substances (i.e. these two substances may be competitively acting via a common pathway). Taken together, our study suggests that sustained attention in children with ADHD could be acutely improved by intake of either l-theanine, caffeine or their combination.

Even though we observed improvements in reaction times (i.e. faster reaction times) of sustained attention tasks with the administration of l-theanine, caffeine and their combination among healthy adults in our previous studies, we did not observe improvements in hit rate or the error rate^26,33^. However, in the present study, l-theanine, caffeine and their combination improved the hit rate, but not reaction times in the Go/NoGo task. These contrasting findings are not surprising given that the performance of any cognitive task is subjected to a speed-accuracy tradeoff^69^. That is, a certain task may have a ceiling or flooring effect when only the accuracy rates are concerned, but may reflect the differences of cognitive function of interest among participants as differences of speed (i.e. reaction times). Conversely, a task with ceiling or flooring effects on reaction times may differentiate participants based on accuracy rates. The Go/NoGo task presented in the current study^51,52^ is a perfect example of the latter, given the task by design limited the time the participants had to react to each stimulus, thereby reflecting the differences in sustained attention among participants as differences in accuracy rates. Others have also observed significant improvements in accuracy rates or hit rates, but not reaction times with the administration of l-theanine and caffeine to healthy adults (e.g. Ref.^25,70^. Therefore, the improvements in hit rates observed with l-theanine, caffeine and their combination in the Go/NoGo task and more importantly the improved d-prime seen with the combination could be considered to reflect underlying improvements of sustained attention.

Despite acute improvements of sustained attention associated with caffeine, several previous studies have raised the concern that intake of caffeine may be associated with increased disinhibition and aberrant behaviors^71–73^. For instance, following administration of 100–400 mg of caffeine to 25 hyperkinetic children in a single-blind placebo controlled crossover trial, teachers and parents reported their children as “more noisy, jumpy and silly”^71^. This notion was supported in the present study. Specifically, intake of caffeine was associated with an overall increase of SSRT (i.e. a deterioration of inhibitory control) compared to placebo, suggesting that intake of caffeine may decrease inhibitory control. We also noted a statistical trend of increase in the SSRT (i.e. a trend of deterioration of inhibitory control) with the intake of l-theanine. Interestingly, with the intake of the combination of l-theanine and caffeine, we observed a statistical trend of reduction of SSRT (i.e. a trend of improvement of inhibitory control) compared to placebo. We emphasize the need to establish this novel, interesting finding in a larger clinical trial. However, the fact that l-theanine–caffeine combination was associated with an improvement in sustained attention, without a concurrent deterioration of inhibitory control per se suggests the superiority of the l-theanine–caffeine combination as a potential therapeutic agent for ADHD as compared with either l-theanine or caffeine.

To our knowledge, the present study is the first to examine the effects of l-theanine–caffeine combination on overall cognition using a standardized validated test battery such as the NIH Cognition Toolbox. However, the improvements we observed in overall cognition composite of NIH Toolbox with l-theanine–caffeine combination is not surprising given that multiple interventional trials and meta-analyses of such trials have suggested that particularly the combination of l-theanine and caffeine seems to improve a variety of cognitive functions^25–27,33,36,37^. For instance, Camfield et al. performed a systematic review and meta-analysis of studies that have examined the cognitive effects of the l-theanine–caffeine combination to suggest that intake of a combination of l-theanine and caffeine seems to improve attention switching and intersensory attention in healthy adults^36^. Similarly, in a controlled crossover trial, Haskell et al. observed significant improvements in memory encoding, particularly in the presence of distractors with the administration of the combination of l-theanine and caffeine as compared to a placebo in healthy adults^27^. Our findings regarding the beneficial effects of particularly the l-theanine–caffeine combination are consistent with these findings. Furthermore, our findings suggest that children with ADHD may experience improvements in not only sustained attention and inhibitory control, but also, overall cognition with the intake of a combination of 2.5 mg/kg body weight of l-theanine and 2.0 mg/kg body weight of caffeine.

Increased background reactivity of DMN while exerting sustained attention and inhibitory control is a salient feature of ADHD^6,11,13^. In this study, l-theanine and caffeine decreased Go/NoGo task-related reactivity of the DMN, possibly indicating that intake of both of these substances may be associated with a reduction in mind wandering in children with ADHD. Similarly, l-theanine, caffeine and their combination decreased reactivity of the DMN while exerting inhibitory control in the Stop-signal task possibly contributing to improved inhibitory control associated with l-theanine–caffeine combination. These findings are consistent with the results of prior electrophysiological and neuroimaging studies. For instance, in two placebo-controlled electroencephalography studies conducted on healthy adults, Gomez-Ramirez observed decreased background α oscillations during an intersensory attention task^35^ and increased task-related α oscillations during a visuospatial attention task^74^ with the intake of 250 mg of l-theanine. In a similar electroencephalography study, Kelley et al.^25^ observed decreased background mean α amplitudes with a combination of 100 mg of l-theanine and 50 mg of caffeine among healthy adults engaged in a cued attention shift task along with concurrent improvements task performance indicative of sustained attention. Corroborating these findings, in a previous fMRI study conducted on healthy adult males, with the intake of l-theanine and l-theanine–caffeine combination, we observed decreased reactivity of the posterior DMN while responding to Go stimuli as well as avoiding NoGo stimuli^33^. Even though we did not observe a significant reduction of task-related DMN reactivity with the intake of l-theanine–caffeine in the present study, given our previous findings, this may have been due to our study being underpowered to detect this mechanism of action. Yet, the fact that l-theanine–caffeine combination was associated with the greatest improvement of hit rate in the Go/NoGo task suggests the possibility of an alternative neurophysiological mechanism besides decreased DMN activity that may be mediating the greater improvement of hit rate associated with the combination of l-theanine and caffeine.

Our study had several limitations that restricts our ability to make robust causal inferences. First, because the objective of the study was to provide preliminary evidence, the study was underpowered to detect a significant improvement in inhibitory control with the administration of the combination of l-theanine and caffeine. While the small sample size is certainly a limitation, the repeated measures design considered four separate measurements from each participant for a given outcome (i.e. 20 total measurements) and yielded sufficient power to replicate several prior observations as well as to result in a trend of improvement of inhibitory control even with five participants. Second, we limited our sample to only male children with ADHD. Furthermore, we limited our sample to children with ADHD who have responded to stimulant medications and are not currently on non-stimulant medications. While this highly selective sample constrained the variance of outcome measures, these strict eligibility criteria also limits our ability to generalize the observed efficacy the l-theanine–caffeine combination to all children with ADHD. Third, the broad ranges of age (i.e. 9–15 years) and BMI (i.e. 14.94–33.29 kg m^−2^) of the participants were limitations, particularly given the rapid changes of neurophysiological and cognitive functions occurring during adolescence and the well-known effects of obesity on brain functional reactivity in ADHD and other developmental disorders^75,76^. Yet, our repeated measures design allowed each participant to act as his own control, limiting the impact of between-subject variability. Fourth, while the single-blinded nature of the study design may have minimally affected the neuroimaging findings and results of Go/NoGo and Stop-signal tasks, the outcomes of NIH Cognition Toolbox, which rely on scoring by study personnel, is not immune to being biased due to unblinding. Fifth, the fact that two of the four administered beverages were bitter while the other two being taste-less (i.e. partial blinding) is a limitation that could have biased the outcomes. Sixth, as in any repeated-measured crossover, practice effect was a limitation. Specifically, the participants were not trained to perform the NIH Cognition Toolbox prior to the testing session. We tried to address this limitation by including the testing visit as a covariate in all analyses and observed a significant effect of study visit on NIH Toolbox cognition composite. Seventh, not providing the participants with standardized food/beverages (except for the test doses) or instructions to fast prior to each testing visit was a limitation, given that perceived level of hunger could affect sustained attention, impulsivity and fMRI reactivity of the brain^77,78^. Finally, caffeine and l-theanine may have an influence on cerebral circulation. Given that fMRI reactivity of the brain depends entirely on regional oxygenated hemoglobin levels, it is not completely clear whether the observed reductions in reactivity of in the brain are entirely due to underlying reductions in neural responses or at least partially due to decreased cerebrovascular responses. However, the task-related reductions of fMRI reactivity in DMN was consistent with increased sustained attention via decreased mind wandering and therefore is unlikely to have been caused by an effect of l-theanine or caffeine on cerebral vasculature.

While our results suggest the potential of l-theanine–caffeine combination to be translated as a pharmacological agent to manage symptoms and cognitive deficits associated with ADHD, future studies should be aimed at answering several important questions. First, the preliminary evidence presented in the current study needs to be replicated in a larger prospective controlled clinical trial that addresses the limitations of the present study. Second, potential differences in therapeutic efficacy based on age and sex needs to be explored. Third, at least caffeine has been shown to be inferior in terms of therapeutic efficacy when compared with stimulants such as methylphenidate in improving ADHD symptoms. Therefore, future studies should aim to compare the therapeutic efficacy of at least the l-theanine–caffeine combination with currently used stimulant medications, in an attempt to justify the use of l-theanine–caffeine combination as an alternative to stimulants. Fourth, on long-term administration, stimulant medications have been suggested to normalize ADHD-related brain structural and functional alterations^79^. Long-term effects of l-theanine–caffeine combination need to be established and compared with the stimulant medications before intake l-theanine–caffeine combination can be recommended as an alternative to stimulants. Fifth, some children with ADHD can develop pharmacological tolerance to currently used medications and others may not respond to the maximum tolerated doses of the medications. Even if l-theanine–caffeine combination appears to be inferior to the currently used stimulants, based on our results, there is a potential to use the combination as an adjuvant to increase the number of stimulant-medication free days to prevent tolerance and to complement the action of currently used medications. Yet, the interactions of l-theanine and caffeine with currently used medications need to be examined. Sixth, based on prior human studies, even much larger doses of l-theanine and caffeine appear to be safe even for long-term administration and likely to be tolerated by a majority^43,44^. However, more studies need to be conducted to assess the risks vs. benefits of treatment with l-theanine and caffeine. Seventh, the plasma half-life of caffeine approximates that of methylphenidate (i.e. ~ 3.5 h), yet the plasma half-life of l-theanine is ~ 1.5 h^41,42^. Therefore, studies need to be conducted to determine the optimal frequency of administration of l-theanine and caffeine. Finally, inappropriate use of ADHD medications (e.g. methylphenidate) to enhance academic and cognitive performance is not uncommon^22,23^ and caffeine is well known to be misused given its role to enhance cognition and arousal^80^. Therefore, recommending the l-theanine–caffeine combination to improve sustained attention and cognition could increase the risk of misuse and also raises the ethical questions ‘is cognitive enhancement with medications or supplements fair and should this be allowed?’^81^. Therefore, while we underscore the potential of l-theanine–caffeine combination to treat ADHD-related cognitive deficits based on our findings, we emphasize the need for further research before l-theanine–caffeine combination can be recommended as an alternative or a complementary treatment for ADHD.

In conclusion, our results suggest that administration of a combination of 2.5 mg/kg of l-theanine and 2.0 mg/kg of caffeine may result in an acute (i.e. short-term) improvements in sustained attention and overall cognition composite of NIH Cognition Toolbox among boys with ADHD, possibly by reversing increased task-related mind wandering associated with ADHD. In addition, our results suggest the potential of the l-theanine–caffeine combination to improve inhibitory control (i.e. decrease disinhibition) among boys with ADHD. While the results of this preliminary study conducted using a small sample size is not sufficient to generalize these benefits, our results emphasize the potential of this naturally occurring combination to be developed into a safe, effective therapeutic strategy to manage ADHD. Larger controlled trials are warranted to confirm this notion and establish additional supportive mechanistic evidence.

## Supplementary information

Supplementary information
