# Supplementary material for: Effects of l-theanine–caffeine combination on sustained attention and inhibitory control among children with ADHD: a proof-of-concept neuroimaging RCT
Source: Sci Rep. 2020 Aug 4;10:13072. doi: 10.1038/s41598-020-70037-7 (PMC7403383; doi:10.1038/s41598-020-70037-7)
Supplement: Supplementary file 1 — Supplementary information [file 41598_2020_70037_MOESM1_ESM.docx]

**Supplemental Methods**

**Title**

Effects of L-theanine-caffeine combination on sustained attention and inhibitory control among children with ADHD: a proof-of-concept neuroimaging RCT

**Authors**

Chanaka N. Kahathuduwa (M.B.B.S., Ph.D.) ^1, 2, 3*^, Sarah Wakefield (MD) ^2, 3^, Blake D. West (B.A.) ^3^, Jessica Blume (M.S.) ^3^, Tharaka L. Dassanayake ^4, 5^, Vajira S. Weerasinghe ^4^, Ann Mastergeorge (Ph.D.) ^3^

**Affiliations**

^1^ Department of Laboratory Sciences and Primary Care, School of Health Professions, Texas Tech University Health Sciences Center, Lubbock, Texas, USA.

^2^ Department of Psychiatry, School of Medicine, Texas Tech University Health Sciences Center, Lubbock, Texas, USA.

^3^ Department of Human Development and Family Studies, College of Human Sciences, Texas Tech University, Lubbock, Texas, USA.

^4^ Department of Physiology, Faculty of Medicine, University of Peradeniya, Peradeniya, Sri Lanka.

^5^ School of Psychology, The University of Newcastle, NSW, Australia.

**Corresponding Author**

Dr. Chanaka N. Kahathuduwa, MBBS, MPhil, PhD,

Assistant Professor, Dept. of Laboratory Sciences and Primary Care (PA Program), School of Health Professions,

Clinical Assistant Professor, Dept. of Psychiatry, School of Medicine,

Texas Tech University Health Sciences Center, 3601 4th Street, Lubbock TX 79430 USA.

Email: [chanaka.kahathuduwa@ttuhsc.edu](mailto:chanaka.kahathuduwa@ttuhsc.edu); Tel: +1-432-620-1120; Fax: +1-432-620-8605

**Order of Administration of Treatments**

| Participant | Intervention Day 1 | Intervention Day 2 | Intervention Day 3 | Intervention Day 4 |
| --- | --- | --- | --- | --- |
| 1 | L-theanine | Combination | Caffeine | Placebo |
| 2 | Combination | Placebo | Caffeine | L-theanine |
| 3 | Placebo | Caffeine | L-theanine | Combination |
| 4 | Caffeine | Combination | L-theanine | Placebo |
| 5 | L-theanine | Placebo | Caffeine | Combination |

**Details of the Administered Cognitive Function Tests**

MRI Scanning Protocol

The fMRI data were acquired using an echo planar sequence defined by the following parameters: TR = 2,140 ms; TE = 25; flip angle = 70˚; FOV = 192 mm X 192 mm; acquisition matrix = 64 X 64; slice thickness = 2.5 mm; and 42 ascending axial slices. The slices were tilted approximately 30˚ from the anterior commissure-posterior commissure line to minimize signal dropout from the orbitofrontal cortex ^1^. The scanning session also included a 4.5-minute T1-weighted structural scan performed using the following parameters: TR = 1,900 ms; TE = 2.49; flip angle = 9˚; FOV = 240 mm X 240 mm; acquisition matrix = 256 X 256; slice thickness = 0.9 mm; and 192 sagittal slices.

Go/NoGo Continuous Performance Task

In the Go/NoGo task ^2, 3^, 12 letters (i.e. A, B, C, F, G, H, K, L, M, O, X, Z) were presented one at a time in a quasi-random order. The letters were presented in black color at the center of a white background. Each stimulus was presented for 500 ms with a 1500 ms ± 1000 ms jittered inter-stimulus interval, depicted by a black fixation cross. A total of 360 trials were presented, in which the letter O (i.e. the cue) was presented in 72 trials. In 36 trials letter X (i.e. the target) was presented subsequent to the cue trials (i.e. Go trials). In the remaining 36 trials following the letter O a letter other than X was presented (i.e. NoGo trials). Letter X was presented in another 36 instances without being preceded by letter O. Letters other than the cue or the target were presented in the remaining 180 trials. The participants were instructed to respond by pressing a response button on a fiber optic hand-held device using the index finger of the right hand, only when they observed letter X immediately after letter O (i.e. only in response to Go trials). Reaction times of all responses, commission errors (i.e. responses to NoGo trials) and omission errors (i.e. Go trials that were not responded) were recorded. The total duration of the task was 12 minutes.

Stop-signal Task

The Stop-signal task, was made-up of a primary Go task and a secondary Stop task ^4^. At the onset of each trial, a black fixation cross was displayed at the center of a white background for a jittered duration of 2500 ms ± 1500 ms, followed by the letter X or letter O appearing at probability of 0.5 in black color. On appearance, each letter remained on the screen for 500 ms. Participants were instructed to press the left response button on a 2-button fiber optic hand-held device using the index finger of the right hand in response to X and the right response button using the middle finger of the right hand in response to O as quickly as possible. In 1/3 of the trials, in a small delay (e.g. 250 ms) of the onset of the letter stimulus (i.e. Stop-signal delay; SSD), the background of the screen was converted to red color, indicating the participant not to respond (i.e. Stop trials). In the remaining trials, the color of the background remained white, indicating the participants to respond (i.e. go trials). After the disappearance of the letter X or O, the background remained for 750 ms and was replaced by a fixation cross, indicating the onset of the next trial. If a participant accurately inhibited the response when the Stop signal was presented, SSD of the subsequent Stop trial was increased by 50 ms. If a participant failed to inhibit the response in the presence of the Stop signal, SSD was decreased by 50 ms. The entire task was made up of 180 trials, of which 120 trials were go trials and 60 trials were Stop trials. Reaction times in go trials, mean SSD throughout the task (known as Stop signal reaction time; SSRT), omission errors in go trials, commission errors in go trials (i.e. responding with the wrong button), commission errors in Stop trials (i.e. responses to Stop trials) were recorded. The total duration of the task was 12 minutes.

NIH Cognition Toolbox

The NIH Cognition Toolbox test battery ^5, 6^ included a flanker inhibitory control and attention test (3 minutes) ^7^, a picture sequence memory test (7 minutes) ^8^, a list sorting working memory test (7 minutes) ^9^, a picture vocabulary test (4 minutes) ^10^, an oral reading recognition test (3 minutes) ^10^, dimensional change card sort test (4 minutes) ^7^ and a pattern comparison processing speed test (3 minutes) ^11^.

MRI Data Pre-processing

Structural MRI data were subjected to brain extraction using Freesurfer (autorecon1) ^12, 13^. Functional MRI data were subjected to following preprocessing steps: motion correction by aligning each functional volume to the center volume within each functional run with 6-DOF sinc interpolation via MCFLIRT ^14^ tool in FMRIB Software Library (FSL, version 6.0, Oxford, UK); skull-stripping using the BET tool in FSL ^15^; registration to high resolution structural space via the BBR algorithm and subsequently to the standard space by 12-DOF using FLIRT tool in FLS ^16^; spatial smoothing using a Gaussian kernel of FWHM 8.0 mm; grand-mean intensity normalization of the entire 4D dataset by a single multiplicative factor; high-pass temporal filtering (Gaussian-weighted least-squares straight line fitting, with sigma = 50.0 s); and FILM pre-whitening ^17^.

Functional MRI Data Analysis

Functional MRI data of the Go/NoGo task were analyzed via two-level models constructed using the FEAT tool in FSL. In level-1 analyses, single task functional time series were convolved on task-based regressors representing onsets of correctly responded Go trials, correctly avoided NoGo trials, and incorrectly responded NoGo trials (i.e. false alarms) using a canonical double gamma function, which closely resembles the hemodynamic response. Contrasts were modeled to capture the correctly responded Go trials, correctly inhibited NoGo trials and false alarm trails. In level-1 analyses of the functional MRI data of the Stop-signal task, single task functional time series were similarly convolved on onsets of correctly responded ‘X’ Go trials, onsets of correctly responded ‘O’ Go trials, correctly inhibited Stop trials and responded Stop trials ^4^. Inhibitory control (i.e. the contrast between the correctly inhibited Stop trials and correctly responded ‘X’ and ‘O’ Go trials) was modeled as a contrast ^4^. Six motion parameters, their temporal derivatives, and regressors to scrub (i.e. censor) volumes that exceeded a frame-wise displacement of 0.9 mm were included in the model as confounders. Serial dependencies between samples not accounted for by the task and confound variables were accounted for via an autocorrelation correction.

In level-2 analyses, level-1 averages and contrasts of the Go/NoGo task and Stop-signal task of all testing visits of all participants were regressed on four variables that represented each of the administered substances (i.e. L-theanine, caffeine, combination and placebo) and five dummy variables that accounted for the within-participant nature of the design using mixed-effects models that considered subject as a random effect for population inference (FLAME 1 and 2). Testing session was included as a confounder to account for the practice effect. L-theanine vs. placebo, caffeine vs. placebo and L-theanine-caffeine combination vs. placebo contrasts were explored within DMN and CEN (defined using publicly available mask templates; <https://figshare.com/articles/fmri_network_templates/2059257>) ^18-20^, which are known to show abnormal reactivity patterns in children with ADHD ^21-23^. Final statistical maps were corrected for multiple comparisons at P < 0.05 using FSL’s permutation-based cluster thresholding (randomise; 5000 permutations; 2.2 t-threshold corresponding to the two-tailed t-threshold of 11 degrees of freedom at significance level of 0.05). The permutation-based approach is robust to inflation of the type I error rates associated with traditional Gaussian Random Field Theory based analysis pipelines ^24^. Atlasquery tool in FSL was used in conjunction with Harvard-Oxford cortical and subcortical structural atlases to localize brain regions showing significant contrasts.

**References**

1. R. Deichmann, J. A. Gottfried, C. Hutton, R. Turner, Optimized EPI for fMRI studies of the orbitofrontal cortex. *Neuroimage* **19**, 430-441 (2003).

2. G. McLoughlin *et al.*, Electrophysiological evidence for abnormal preparatory states and inhibitory processing in adult ADHD. *Behav. Brain. Funct.* **6**, 66 (2010).

3. G. McLoughlin *et al.*, Cognitive-electrophysiological indices of attentional and inhibitory processing in adults with ADHD: familial effects. *Behav. Brain. Funct.* **7**, 26 (2011).

4. A. D. Chevrier, M. D. Noseworthy, R. Schachar, Dissociation of response inhibition and performance monitoring in the stop signal task using event-related fMRI. *Hum. Brain Mapp.* **28**, 1347-1358 (2007).

5. P. D. Zelazo, P. J. Bauer, *National Institutes of Health Toolbox cognition battery (NIH Toolbox CB): Validation for children between 3 and 15 years*. (Wiley, 2013).

6. P. D. Zelazo *et al.*, NIH Toolbox Cognition Battery (CB): Validation of executive function measures in adults. *J. Int. Neuropsychol. Soc.* **20**, 620-629 (2014).

7. P. D. Zelazo *et al.*, II. NIH Toolbox Cognition Battery (CB): measuring executive function and attention. *Monogr. Soc. Res. Child Dev.* **78**, 16-33 (2013).

8. P. J. Bauer *et al.*, III. NIH Toolbox Cognition Battery (CB): measuring episodic memory. *Monogr. Soc. Res. Child Dev.* **78**, 34-48 (2013).

9. D. S. Tulsky *et al.*, V. NIH Toolbox Cognition Battery (CB): measuring working memory. *Monogr. Soc. Res. Child Dev.* **78**, 70-87 (2013).

10. R. C. Gershon *et al.*, IV. NIH Toolbox Cognition Battery (CB): measuring language (vocabulary comprehension and reading decoding). *Monogr. Soc. Res. Child Dev.* **78**, 49-69 (2013).

11. N. E. Carlozzi, D. S. Tulsky, R. V. Kail, J. L. Beaumont, VI. NIH Toolbox Cognition Battery (CB): measuring processing speed. *Monogr. Soc. Res. Child Dev.* **78**, 88-102 (2013).

12. A. M. Dale, B. Fischl, M. I. Sereno, Cortical surface-based analysis. I. Segmentation and surface reconstruction. *Neuroimage* **9**, 179-194 (1999).

13. B. Fischl *et al.*, Sequence-independent segmentation of magnetic resonance images. *Neuroimage* **23 Suppl 1**, S69-84 (2004).

14. M. Jenkinson, P. Bannister, M. Brady, S. Smith, Improved optimization for the robust and accurate linear registration and motion correction of brain images. *Neuroimage* **17**, 825-841 (2002).

15. S. M. Smith, Fast robust automated brain extraction. *Hum. Brain Mapp.* **17**, 143-155 (2002).

16. M. Jenkinson, S. Smith, A global optimisation method for robust affine registration of brain images. *Med. Image Anal.* **5**, 143-156 (2001).

17. M. W. Woolrich, B. D. Ripley, M. Brady, S. M. Smith, Temporal autocorrelation in univariate linear modeling of FMRI data. *Neuroimage* **14**, 1370-1386 (2001).

18. V. D. Calhoun, T. Adali, G. D. Pearlson, J. J. Pekar, A method for making group inferences from functional MRI data using independent component analysis. *Hum. Brain Mapp.* **14**, 140-151 (2001).

19. C. Chang, G. H. Glover, Effects of model-based physiological noise correction on default mode network anti-correlations and correlations. *Neuroimage* **47**, 1448-1459 (2009).

20. M. E. Thomason *et al.*, Resting-state fMRI can reliably map neural networks in children. *Neuroimage* **55**, 165-175 (2011).

21. A. Christakou *et al.*, Disorder-specific functional abnormalities during sustained attention in youth with attention deficit hyperactivity disorder (ADHD) and with autism. *Mol. Psychiatry* **18**, 236 (2013).

22. R. A. Barkley, Behavioral inhibition, sustained attention, and executive functions: constructing a unifying theory of ADHD. *Psychol. Bull.* **121**, 65 (1997).

23. D. Van Rooij *et al.*, Neural activation patterns in inferior frontal areas during response inhibition distinguish adolescents with ADHD, their unaffected siblings, and healthy controls. *Am. J. Psychiatry*, (2014).

24. A. Eklund, T. E. Nichols, H. Knutsson, Cluster failure: Why fMRI inferences for spatial extent have inflated false-positive rates. *Proc. Natl. Acad. Sci. U. S. A.* **113**, 7900-7905 (2016).
